# Effectiveness of behavior change interventions for smoking cessation among expectant and new fathers: findings from a systematic review

**DOI:** 10.1186/s12889-023-16713-5

**Published:** 2023-09-18

**Authors:** Sudeepa Khanal, Céline Miani, Emily Finne, Julia Zielke, Melanie Boeckmann

**Affiliations:** 1 Department of Epidemiology & International Public Health, School of Public Health, Bielefeld University, Universitätsstraße 25, 33615 Bielefeld, Germany; 2 Department of Global Health, Institute of Public Health and Nursing Research, University of Bremen, Bremen, Germany

**Keywords:** Behavioral change intervention, Smoking cessation, Expectant and new fathers, Gender targeted intervention, Systematic review

## Abstract

**Background:**

Smoking cessation during pregnancy and the postpartum period by both women and their partners offers multiple health benefits. However, compared to pregnant/postpartum women, their partners are less likely to actively seek smoking cessation services. There is an increased recognition about the importance of tailored approaches to smoking cessation for expectant and new fathers. While Behavior Change Interventions (BCIs) are a promising approach for smoking cessation interventions, evidence on effectiveness exclusively among expectant and new fathers are fragmented and does not allow for many firm conclusions to be drawn.

**Methods:**

We conducted a systematic review on effectiveness of BCIs on smoking cessation outcomes of expectant and new fathers both through individual and/or couple-based interventions. Peer reviewed articles were identified from eight databases without any date or language restriction.Two independent reviewers screened studies for relevance, assessed methodological quality of relevant studies, and extracted data from studies using a predeveloped data extraction sheet.

**Results:**

We retrieved 1222 studies, of which 39 were considered for full text screening after reviewing the titles and abstracts. An additional eight studies were identified from reviewing the reference list of review articles picked up by the databases search. A total of nine Randomised Control Trials were included in the study. Six studies targeted expectant/new fathers, two targeted couples and one primarily targeted women with an intervention component to men. While the follow-up measurements for men varied across studies, the majority reported biochemically verified quit rates at 6 months. Most of the interventions showed positive effects on cessation outcomes. BCI were heterogenous across studies. Findings are suggestive of gender targeted interventions being more likely to have positive cessation outcomes.

**Conclusions:**

This systematic review found limited evidence supporting the effectiveness of BCI among expectant and new fathers, although the majority of studies show positive effects of these interventions on smoking cessation outcomes. There remains a need for more research targeted at expectant and new fathers. Further, there is a need to identify how smoking cessation service delivery can better address the needs of (all) gender(s) during pregnancy.

**Supplementary Information:**

The online version contains supplementary material available at 10.1186/s12889-023-16713-5.

## Background

Tobacco smoking during pregnancy poses substantial health risks to both mother and child. Maternal smoking is the most significant preventable cause of serious complications in pregnancy, including low birthweight, preterm birth, stillbirth and neonatal death. Second-hand smoke (SHS), also called passive smoking or environmental tobacco smoke, is a mixture of smoke exhaled by smokers and smoke released from smoldering cigarettes, cigars, pipes, bidis, etc. The smoke mixture contains gases and particulates, including nicotine, carcinogens, and toxins [1]. SHS is estimated to have caused about 603,000 premature deaths worldwide in 2004 with 28% and 47% of the attributable deaths being among children and women respectively [1]. SHS is more harmful to unborn children than women smoking themselves [1]. Studies have noted the association between SHS exposure and negative birth and fetal health outcomes including stillbirth, congenital malformation [2] and low birth weight [3]. Tobacco smoke exposure during prenatal and postnatal lung development contributes to respiratory morbidities during childhood [4, 5]. SHS is also associated with an increased risk of developing lung cancer, coronary heart disease and stroke in adult non-smokers [6, 7]. Fathers are one of the main sources of SHS for pregnant women, resulting in various types of congenital health defects (CHD) in offspring. A meta-analysis of data from 125 studies involving more than 100,000 children with CHDs, indicated that parental smoking was significantly associated with risk of CHDs, with an increased risk of 25% for maternal active smoking, 124% for maternal passive smoking and 74% for paternal active smoking, compared with non smokers [8].

Smoking cessation during pregnancy and the postpartum period by both women and their partners offers multiple health benefits [9, 10] and have become an integral part to maternal and child public health policies [11]. However, there are many different reasons why expectant and new parents struggle to quit smoking. Various social, physiological, psychological, and behavioral factors shape smoking cessation efforts [12, 13]. Sociocultural influences have also been identified as dominant barriers to achieving effective cessation outcomes. There are strong linkages between smoking behavior (in general and) during pregnancy and postpartum, and role of partner support for successful quit attempts [14] and reduction in postpartum relapse rates in pregnant women [15, 16]. To achieve positive cessation outcomes, continued abstinence, and to prevent relapse in women during and after pregnancy, it is important to consider the role of partners and their own smoking behavior. It is also important to note that tobacco control programmes generally assume heterosexual couples to be the default norm and partners to be men [17, 18]. Most of the literature addressing pregnant women partners smoking refers exclusively to male partners, and biological fathers and only few interventions are inclusive of sexual and gender minorities (SGM) [19, 20]. Considering this bias in practice and research and the strong role of masculine gender norms in relation to both smoking and parenthood, we focus our review on men and fathers.

Gender is indeed a key determinant of smoking and a core socio-cultural factor underpinning smoking behavior [21]. Traditional gender norms related to tobacco-use position smoking behavior as an expression of masculinity and associate female smoking with misconduct and social stigma, thus playing an important role in one’s decision to both initiate and quit smoking [22]. Traditional societal notions surrounding masculinity and femininity also influence men's and women’s expectations about their new roles as they prepare to become parents. Traditional gender views often set certain standards for parents and not being able to fulfil those may result in judgment or stigmatisation. Men are still often expected to be the provider of the family and women seen as primary caretakers of the child [23]. These gendered stances on parenting including stereotypes of what being “good parents” means, may encourage both expectant parents to make positive changes in their health behavior, including attempting to quit smoking [9, 24]. First-time fathers are noted to be more receptive to smoking cessation support or to modifying their own smoking behavior early in their partner’s pregnancy [25]. Antenatal care (ANC) is a prime opportunity to engage with partners of pregnant women and provide them with smoking cessation support. Nonetheless, the extent of engagement with partners on the topic of smoking cessation during Antenatal care sessions varies across different context and settings [26].

Behavioral Change Interventions (BCIs) are useful approaches to smoking cessation as they include relevant aspects such as motivation, self-efficacy, consideration of barriers and benefits to change, subjective norms, attitudes, and socio-cultural factors [27]. Various definitions have been proposed for BCIs [27–30]. In this review, we apply the definition proposed by Michie and Johnston [31] who define BCIs as inteventions that include one or more Behavior change Techniques (BCTs). They describe a BCT as “a systematic procedure included as an active component of an intervention designed to alter behavior”, with the defining characteristics of BCTs being observability, replicability,, irreducibility, incorporation of a behavior change components, and a postulated active ingredient within the intervention.

Even though various systematic reviews have assessed the efficacy of BCIs on smoking cessation outcomes of expectant parents, both as multi-strategic as well as single interventions [32], most available evidence relates to interventions tailored to pregnant women and concludes to varying levels of success [24, 30, 33, 34]. Many of the studies explored (expectant and/or new) fathers smoking only as a facilitating or inhibiting factor for pregnant women to quit. For example, a Cochrane systematic review conducted by Chamberlin et al. (2017) on smoking cessation interventions for pregnant women, excluded studies with interventions aimed at partners [30]. This review however, discussed the importance of fathers smoking cessation to achieve positive smoking cessation outcomes among pregnant women. In the context of smoking cessation programs for expectant and new fathers, several theories suggest that couple‐focused interventions for health behavior change may be more effective than individual interventions in facilitating long‐term maintenance [35]. However, there is no systematic review specifically on the effectiveness of BCIs on partner smoking and partner cessation [14, 36–38] nor on behavior change strategies for smoking cessation for men with a focus on pregnancy and postpartum. From our initial search, we identified one systematic review exploring the efficacy of gender- specific strategies for smoking cessation. That study focussed on men and women between 40–65 years of age in the general population [39]. Similarly, we located another qualitative systematic review that explored the barriers and facilitators to smoking cessation experienced by women’s partners during pregnancy and the post-partum period. This review however did not investigate the effectiveness of (BCI) interventions but focused on partners’ perceptions and experiences of smoking cessation during and after pregnancy [40]. Against this background, we conducted a systematic review to explore if any and what types of effects can be expected from behavior change-focused smoking cessation interventions tailored to male partners of pregnant and postpartum women.

### Rationale

Despite the accumulating evidence on a) harmful effects of SHS during pregnancy, b) identified needs to support expectant and new fathers to quit smoking and c) potential benefits of partner support for pregnant women quitting smoking, fewer studies have investigated the effectiveness of smoking cessation interventions for expectant and new fathers. Overall, BCIs seem a promising approach to support smokers to address smoking behavior. Whether this applies to the target group of expectant and new fathers remains to be examined. Also,information on how to best design BCIs specifically for expectant and new fathers is fragmented.

This review systematically examines the existing evidence on effectiveness of BCIs on smoking cessation outcomes of expectant and new fathers, both through individual and/or couple-based interventions. To the best of our knowledge, this is one of the few formal attempts to examine the effectiveness of BCIs for smoking cessation in pregnant couples and new parents using a gendered lens.

### Objectives

The primary objective of this review was to systematically determine the effectiveness of BCIs for smoking cessation when given to the partners of pregnant women and new mothers (expectant and new fathers).

The secondary focus of this review was to explore various BCI used to date to address expectant and new fathers’ smoking behaviour.

### Review questions

The review questions for the study were:

- What is the effectiveness of BCIs for smoking cessation in expectant and new fathers?
- How does the effectiveness of BCIs compare between a) specifically targeted to expectant and new fathers and b) when given as an add-on component to interventions targeted at pregnant women or postpartum mothers?

Drawing from the psychological literature that defines the transition to fatherhood as the period from conception to one year after birth, for this review, we define “new fathers” as fathers until first year postpartum [41].

## Materials and methods

**Fig. 1:**
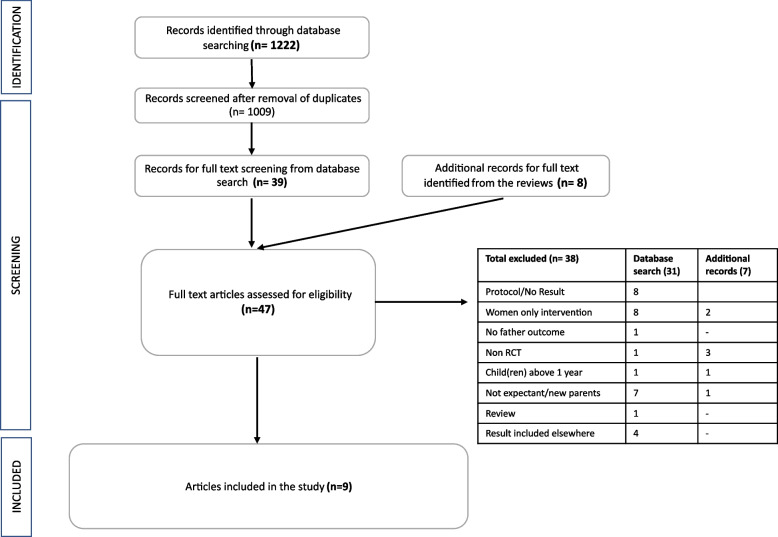
PRISMA flow Diagram

This systematic review was conducted according to the Preferred Reporting Items for Systematic Reviews and Meta-analyses (PRISMA) guidelines [42] and reported accordingly (Fig. 1). The protocol was registered in PROSPERO, the International Prospective Register of Systematic Reviews (CRD42021272213).

### Search terms and strategies

Search terms and strategies were developed in consultation with a research librarian and informed by previous systematic reviews on effectiveness of BCIs in general. An initial search strategy was developed in PubMed and terms were adapted for use in other databases. Eight electronic databases (PubMed, EMBASE, Wiley Online Library, JSTOR, Web of Science Core Collection, APA PsycINFO, Cochrane Central Register of Controlled Trials (CENTRAL) and MEDLINE) were searched without any timeline, language, or geographical restriction (Supplementary material S1). The study search were conducted on November 2021, corresponding to the timeline of the project. In addition, the reference lists of the identified reviews on BCIs for smoking cessation among men were cross-checked to identify additional relevant studies not detected by the original literature search.

### Study selection

For inclusion, the study had to be a) a randomized control trial (RCT) assessing the effects of BCIs on smoking outcomes among expectant and new fathers (with child(ren) below 1 year of age) or b) RCT assessing interventions on pregnant women and/or new mothers (with child(ren) under 1 year of age) with a component of cessation support to partners. Studies had to include expectant and new fathers who smoked during the time of intervention irrespective of their level of nicotine dependence, intention to quit or predetermined physical and mental conditions. No restriction was applied regarding intervention duration, setting or mode of delivery. RCTs exclusively reporting on any alternatives/substitutes to smoking cigarettes (cannabis, electronic nicotine delivery systems (ENDS), also called electronic cigarettes or e-cigarettes) only were excluded. Reviews were excluded. As mentioned above, BCI was defined as per the definition proposed by Michie and Johnston and included a range of interventions with different a) treatment format, b) approach, c) mode of delivery, d) number of sessions e) method and f) delivery setting [31].

### Data management and extraction

All the records from scientific databases were imported to EndNote(X9) and duplicate citations removed. Screening was conducted by two independent reviewers (SK and CM) for inclusion in two stages based on predefined inclusion criteria (stage 1: titles and/or abstracts, stage 2: full text). In the full text screening, log of the excluded studies was kept stating the reason for exclusion. In case of disagreement, a decision of eligibility for inclusion were resolved by discussion between the two reviewers or consultation with the third reviewer (MB). Data were extracted into a pre-defined structured template by two reviewers (SK and CM), compared and agreed on.

### Critical appraisal

Quality assessment was conducted by three independent reviewers (SK, CM and MB) using the Revised Cochrane risk-of-bias tool for randomized trials (RoB 2) checklist [43]. A check among researchers for consistency of quality assessment was conducted after completion of the initial 3 studies, and one researcher (SK) compared the assessment results at the end of data extraction. Discrepancies were resolved by consensus among all reviewers.

## Results

### Literature search

The initial database search produced 1222 scientific articles. After excluding 213 duplicates,1009 abstracts were accessed for eligibility. As shown in the PRISMA flow diagram (Fig. 1), a further 970 articles were excluded following title or abstract review and 39 articles were considered for full text. An additional 8 studies were identified from reviewing the reference list of review articles picked up by the databases search. Finally, a total of nine studies were included in the review. For protocols and abstracts without study results, an email request was sent to authors requesting the trial findings, however, this did not elicit any positive response.

### Critical appraisal

Out of the nine studies in total, six were considered to have a low risk of bias, two had some (minor) concerns and one study was considered to have high risk in the overall assessment (Supplementary material S2).

### Study characteristics

All the nine studies meeting the inclusion criteria were published in the 2000s, with data collection of two studies conducted in the 1990s [44, 45]. Four studies were conducted in China [46–49], three in the USA [44, 50, 51], one in Finland [45] and one in Australia [52]. All included studies were RCTs with three 3-arm RCT [44, 48, 49] and one pragmatic RCT [47]. Pragmatic RCT refers to RCTs that seek to assess the effectiveness of interventions within a ‘real-world scenario’ or a diverse ‘real-world population,’ rather than within a predefined patient group with similar baseline characteristics that impact prognosis, as seen in stratified RCTs. They intend to produce evidence directly applicable to patients, caregivers, and healthcare system managers for informed policy-making and choices [53].

The focus of eight studies interventions were specifically on smoking cessation, whereas one included smoking cessation only as a component of wider lifestyle modification [45]. Six studies primarily aimed to assess effectiveness of the intervention to increase quit rates specifically among expectant and new fathers [46–49, 51, 52], two were parent-centered intervention [45, 50] for creating smokefree homes/ reducing SHS for children and one assessed fathers’ smoking as a component while addressing abstinence rates among pregnant women during and after pregnancy [44].

### Settings and participant recruitment methods

All the nine studies were conducted primarily in healthcare setting and men were identified and recruited through their partners. The studies featured a wide range of health care facilities ranging from maternal child health centers, antenatal/prenatal clinics, medical centers to rural and urban county health departments and child clinics. Five studies [44, 47, 48, 51, 52], recruited participants from antenatal clinics during prenatal appointments. One trial [45] recruited families of 6-month-old infants from a Cardiorespiratory Research Unit, one study [46] recruited parents of newborns after delivery at the hospital, one study [49] recruited at maternal and child health centers, and another study recruited mothers attending their initial post-delivery visit [50]. None of the studies enrolled participants from the general population. Participant recruitment time ranged between 1 to 3 years, except in two studies with recruitment duration of 1 month and 6 years respectively [44, 49]. The number of health care centers involved in individual studies ranged from 1 to 22.

The nine studies collectively included 6231couples. Of these, 2663 couples were new parents and 3568 were expecting couples. All participating fathers were current smokers, the participating mothers sample included smokers, nonsmokers and smokers who quit recently.

Details of the study characteristics and interventions are shown in Table 1.

**Table 1:** Study characteristics and interventions

Study	Design	Country	Setting	Participants	Mode of Delivery	Intervention description	Time of intervention
Chan et al., 2017 [46]	Parallel 2-arm RCT	Hongkong, China	Maternal and child health centres	1158 families (non-smoking mothers with a neonate aged 0–18 month and a smoking partner	Selfhelp materials, face to face counselling	Mothers – onsite counselling sessions, 2 self-help booklets on smoking cessation and maintaining a smoke-free home; and a card specifying the follow-up schedule from the nurse counsellor, 2 telephone counselling sessions at 1 week and 1 month, advice to help fathers quit and establish a smokefree home to reduce infants’ SHS exposure. Fathers-3 telephone counselling sessions at 2 days, 1 week, and 1 month (30 min), advice to quit smoking. 1 week of free Nicotine Replacement Therapy (NRT) and some incentives for participation. In addition to the individual counselling, the father and mother voluntarily participated in a family counselling session (FCS) aimed at establishing mutual support, encouraging effective discussion, and setting goals for smoking cessation Control: Fathers:no advice on cessation at baseline or any follow-up	Postnatal period
Kallio et al., 2006 [45]	RCT	Finland	Well-baby clinics	1062 infants recruited at the age of 7-month-old and their parents	Selfhelp materials, telephone counselling	Individualized and detailed child-targeted lifestyle counselling at each visit given to the families (at least twice a year), Intervention comprised dietary counselling aimed at reducing the intake of saturated and total fat and cholesterol in the child’s diet. Other major cardiovascular risk factors, including smoking, were discussed with the parents. At the child’s age of 5 y, parents received a booklet about the adverse health effects of smoking. If the family history was positive for premature heart disease, the importance of quitting smoking was repeatedly discussed with the intervention parents Control: normal health education given to all Finnish families at the well-baby clinics	Postnatal period
Luk et al., 2021 [47]	Pragmatic RCT	China	Prenatal clinics in seven public hospitals	1053 smoking partners of pregnant women	Selfhelp materials, telephone and face to face counselling	Expectant fathers received Brief Advice, NRT sampling, and Active Referral (BANSAR) according to the AWARD model Ask: asked expectant fathers at the clinics about their smoking behaviors. Warn: Current cigarette smokers were then invited to test their exhaled CO level and readings shown to the smokers as a warning about the health risks of SHS exposure for pregnant women, the fetus, and young children Advise: The smokers advised to quit smoking as soon as possible and to enroll in the study 1-week sample of NRT patch or gum in its original packaging. Instruction card provided that described how to use the NRT patch or gum and encouraged the participants to make quit attempts without the pressure of quitting successfully. Refer: referral to a free community-based smoking cessation service of their choice that provided evidence-based cessation treatment, including counselling and full-course pharmacotherapy as appropriate. Do it again: leaflet about available services and were encouraged to select a service. Telephone numbers of those who agreed to be referred were sent to the practitioner at the selected service, who subsequently contacted the participants for further treatment. Additionally, 2 telephone boosters (2 min each) within the first month of enrolment to address any issue related to the NRT and the smoking cessation service The pregnant women did not receive any intervention except advice on the health risks of perinatal passive smoking Control: Participants received only brief cessation advice (Ask,Warn, Advise) with a standard leaflet on the hazards of perinatal exposure to tobacco smoke and the toll-free quitline telephone number in Hong Kong. Through the quitline, participants could access the same smoking cessation services to which participants in the intervention group were actively referred	Antenatal period
McBride et al., 2004 [44]	A 3-group RCT	USA	Medical Centre	583 pregnant women and their partners (488 partners eligible)	Selfhelp materials, telephone counselling and video	Participants randomized to usual care (UC), woman-only (WO), or partner-assisted (PA) intervention UC (Control): Women received provider advice to quit smoking at the first prenatal visit and were mailed the self-help guide, written at the fifth-grade reading level and designed for pregnant women WO: Women received UC components plus a late pregnancy relapse-prevention kit (a booklet and gift items) and six counselling calls PA: Women received the WO intervention plus a PA adjunct, in which the smoker described how her partner could be a coach to build and maintain the confidence she needed to quit smoking. Intervention objectives were to (1) encourage couple communication about helpful and unhelpful support behaviors, (2) assist partners in developing alternatives to negative behaviors, (3) prompt couples to make plans for handling high-risk situations, and (4) when appropriate, encourage and assist partner smoking cessation. An “It Takes Two” booklet and companion video were developed to guide couples in discussing support behaviors related to the woman’s smoking. These skills were reinforced during counselling calls Partners received six separate calls- guided by a motivational interviewing protocol. The second and fourth calls to the couple focused on developing a written agreement regarding helpful partner support behaviors. Partners who smoked were given self-help cessation guides, free nicotine patches if needed, and stages (of change) appropriate counselling	Antenatal and postnatal period
Pollak et al., 2014 [51]	RCT	USA	10 urban and rural county health departments	348 expecting couples (non-smoking mothers and smoking partner)	Selfhelp materials, telephone and face to face counselling	Compared (i) written materials plus NRT (less intensive)to [43] materials, NRT, and couple-based counseling that addressed smoking cessation and couples communication (more intensive) Men randomized to the intensive intervention arm received the booklet, an option of up to 6 weeks of NRT, three counselling sessions during pregnancy (one face-to-face and two via phone) and three postpartum (one face-to-face and two via phone) to help them identify and achieve their goals for quitting smoking and improve their communication with their partner. Individual male and female counsellors, counselling with father- build motivation to quit, identify barriers to quitting, and to set a goal to quit or move toward quitting during the pregnancy, counselling with mother- to identify whether she wanted to work on nutrition or physical activity, build motivation for change, identify barriers to change, and to set realistic goal to work toward during pregnancy. Effective communication skills for couples in each session to understand each other's barriers to changing their health behaviors and problem-solve on how to support each other in making these changes Control: Men were given a smoking cessation booklet and an option of up to 6 weeks of NRT	Antenatal and postnatal period
Stanton et al., 2004 [52]	Stratified RCT	Australia	Public antenatal clinic at two large metropolitan hospitals	561 smoking partners of pregnant women	Selfhelp materials, video	After baseline interview men were sent the intervention materials including a letter explaining the study to be taken to their own GP. Video: an 18-min video introduced by a national football personality focusing on becoming a father and on passive smoking health risks for the newborn Nicotine patches and information pack: 1 week’s supply of patches, booklets, and cassette tape on how to use the patches and a booklet on quitting was made available following a telephone assessment by a general practitioner (GP) with explanation of side effects and precautions One week and one month later, package was sent containing support material: newsletter with a reminder on how to use the multicomponent package, tips on quitting, motivational anecdotes and stickers Control: Men were sent a brochure providing contact details for the available smoking cessation options	Antenatal period
Winickoff et al., 2010 [50]	Pilot RCT	USA	Hospital childbirth centre	101 smoking couples	Selfhelp materials, face to face counselling	Intervention condition received one 15-min in-person counselling session from adapted materials and messages specifically tailored for parental smokers, offer of enrolment in a proactive state-of-the-art telephone counselling intervention and letters faxed to the newborn’s pediatrician, parents’ primary care provider, and mother’s obstetrician indicating the parent’s tobacco use status and readiness to quit and recommending useful strategies to facilitate parental cessation, the need for ongoing support, and medication prescription when appropriate Control: Parents who were assigned to the control condition had no contact between the baseline and follow-up surveys	Postnatal period
Xia et al., 2020 [48]	3-arm RCT	China	Obstetrics registration centres of 3 tertiary public hospitals in 3 major cities	1023 smoking expectant fathers	Selfhelp materials, video and text messages	Video group: 4 videos on various risks of smoking for maternal and child health via WeChat. 1 video sent to each participating in weeks 1, 3, 5, and 7. Video content developed using the theory of planned behavior. Each video lasted approximately 1 min, with content focusing on different hazardous effects of smoking on pregnant women, fetuses, and newborns (S2 Text) Text group. Participant fathers received 4 text messages with content similar to that of the videos and on similar schedules (S2 Text) Control: Following receipt of the leaflet at baseline, participants received no further intervention	Antenatal period
Yu et al., 2017 [49]	3-arm RCT	China	15 local maternal-child health centers	342 households underwent randomisation (non-smoking mothers and their smoking partners)	Selfhelp materials, face to face counselling and text messages	Group I-A: in-person counseling on the harms of SHS to infants; education on establishing a smoke-free home, including a manual with step-by-step instructions; and table tents and posters to display in the home to encourage fathers and other visitors not to smoke. The smoke-free homes manual provided a 5-step plan for creating a smoke-free home with information on: (1) deciding to create a smoke-free home; (2) talking to family members; (3) setting a date for going smoke-free; (4) actually creating a smoke-free home; and (5) keeping the home smoke-free Group I-B: same educational intervention and materials as I-A at this visit, and a text message intervention in the coming months. The text message intervention included messages to the mother and her husband on the harms of SHS to the mother and the infant. The husband received additional cessation text messages to encourage him to quit smoking. A total of 9,500 messages were sent to participants in I-B Follow-up home visits were performed at 6 and 12 months. I-A and I-B received additional counselling at the six-month follow-up visit if they had been unable to successfully create a smoke-free home since the initial visit Control: standard care for their initial postnatal visits, which did not include any tobacco control and cessation counseling service	Postnatal period

### Primary target of the interventions

Out of nine included studies, six focused their intervention on men [46–49, 51, 52], two on couples [45, 50] and one on women [44]. Among the six studies addressing men, three clearly highlighted the need of smoking cessation support for expectant and new fathers and exclusively directed their intervention to them [47, 48, 52]. Three other studies [46, 49, 51] primarily addressed men with added information/advice to women. The intervention given to women mostly emphasized the importance of their role for facilitating their partner’s smoking cessation efforts and keeping the house smokefree for the benefit of the health of their child. In these studies, the male partners received more frequent and longer treatment than the included mothers. Two interventions [45, 50] offered the same intervention to couples together. One study [44] mainly focused on women’s cessation and involved partners to promote favorable cessation outcomes in women.

### Providers and intervention delivery mechanisms

Healthcare professionals delivering the intervention varied across studies and included nurses, research nurses/assistants, pediatricians and dietitians, health advisors, trained health workers, and general practitioners. Three studies used more than one provider for different components of the intervention [41, 43, 46]. The participants’ level of interaction with the intervention providers also varied highly across the studies with some studies reporting to have minimal direct contact with participants [44, 48]. Four studies mentioned training the intervention providers, however, details of the training are not reported consistently across the studies [42, 45–47].

The reviewed studies also contained various intervention modes of delivery (MoD) including a booklet/self-help material, telephone counselling, face to face counselling and web or phone-based video or text messaging. In most cases, more than one MoD was adopted and none of the interventions reported to have relied on a single method. Use of self-help materials or booklets was the most frequently used MoD and used by all the interevntions (*n* = 9). This was followed by counseling (*n* = 7) [44–47, 49–51], either face to face (*n* = 5), telephone counseling (*n* = 4), or a combination of both (*n* = 2). Two of the studies [49, 51] provided face to face counseling at couples’ homes. In one study [51], home visit was in addition to telephone counseling and provision of self-help materials. Optional referral to community based smoking cessation services occurred only in two studies [47, 50]. Four studies used digital MoD of interevntion with two using videos [44, 52], one using text messages [49], and one using both videos and text messages via mobile messaging application [48]. One study [50] also provided an optional web based cessation program, the details of which haven’t been mentioned in the paper.

### Length and frequency of intervention

Interventions lasted from one month to repeated sessions in the course of eight years. The length of each session depended on the type and nature of intervention. For example, counselling sessions (either in person or telephone) were slightly longer than follow-up or booster telephone calls. Likewise, the number of sessions and frequency of intervention provision also varied considerably across the studies and were not comparable.

### Control groups

In three studies, the control group consisted of usual care or standard care comprising of a) normal health education given to all families at the child clinics and counselling for school-aged children [45], b) provider advice to quit smoking for women at the first prenatal visit and a self-help guide designed for pregnant women [44] and c) standard care for families in the initial postnatal visits, which did not include any tobacco control and cessation services [49]. Two studies [48, 50] involved baseline and endline surveys with fathers with one of them reporting to have no contact in between with the fathers [50] and the other, gave a leaflet to the parents at baseline [48].

In four other studies, the control group received a leaflet or information booklet in combination with other components. Men were sent a brochure providing contact details of the available smoking cessation options [52], a smoking cessation booklet and an option of up to 6 weeks of NRT [51], brief cessation advice with standard leaflet and toll-free quitline telephone number [47], or a self-help smoking cessation pamphlet for the smoking fathers [46]. The control group in one study [46] also included mothers who received a 2-page leaflet about the importance of establishing a smokefree home as well as brief advice provided by a trained nurse.

### Theoretical underpinnings

All interventions were assessed to identify any explicit or implicit mention of theories, models, or standard guidelines. Across studies, a total of five theories and three models informed the interventions. The extent of use of these theories and models to inform the intervention design was unclear and various terms like “reference”, “based on”, “guided by”, “adapted from” and “drew on” were used. Four studies [46, 48, 50, 51] cited at least one theoretical construct for the intervention design of which two studies reported more than one theory [46, 50]. The most common theories referred were Social Cognitive theory [46, 51] and the Transtheoretical Model of Behavior Change [46, 50]. Other theories and models mentioned were social ecological theory [46], social learning theory [50] and theory of planned behavior [48]. None of the studies described in detail how the theories were used to inform the intervention content.

In terms of the models, AWARD (Ask, Warn, Advise, Refer, and Do it again) model [47], teachable moment model [46] and the health belief model [50] were reported to inform the study interventions. Two of the interventions based their counseling on motivational interviewing [50, 51], of which one provided a 40-h training to the counsellors [51]. None of the studies applied gender specific theories or analytical framework to inform their intervention design.

### BCT analysis

None of the studies explicitly reported the BCTs included in the interventions. Each intervention was therefore coded by authors to identify BCTs in line with the BCT taxonomy (v1) [29]. Only the BCTs that were recognizably included in the intervention provided to fathers were coded. One of the authors (SK) coded each intervention. The coding was reviewed by two other authors (EF, MB) independently and the differences discussed to reach consensus.

**Fig. 2:**
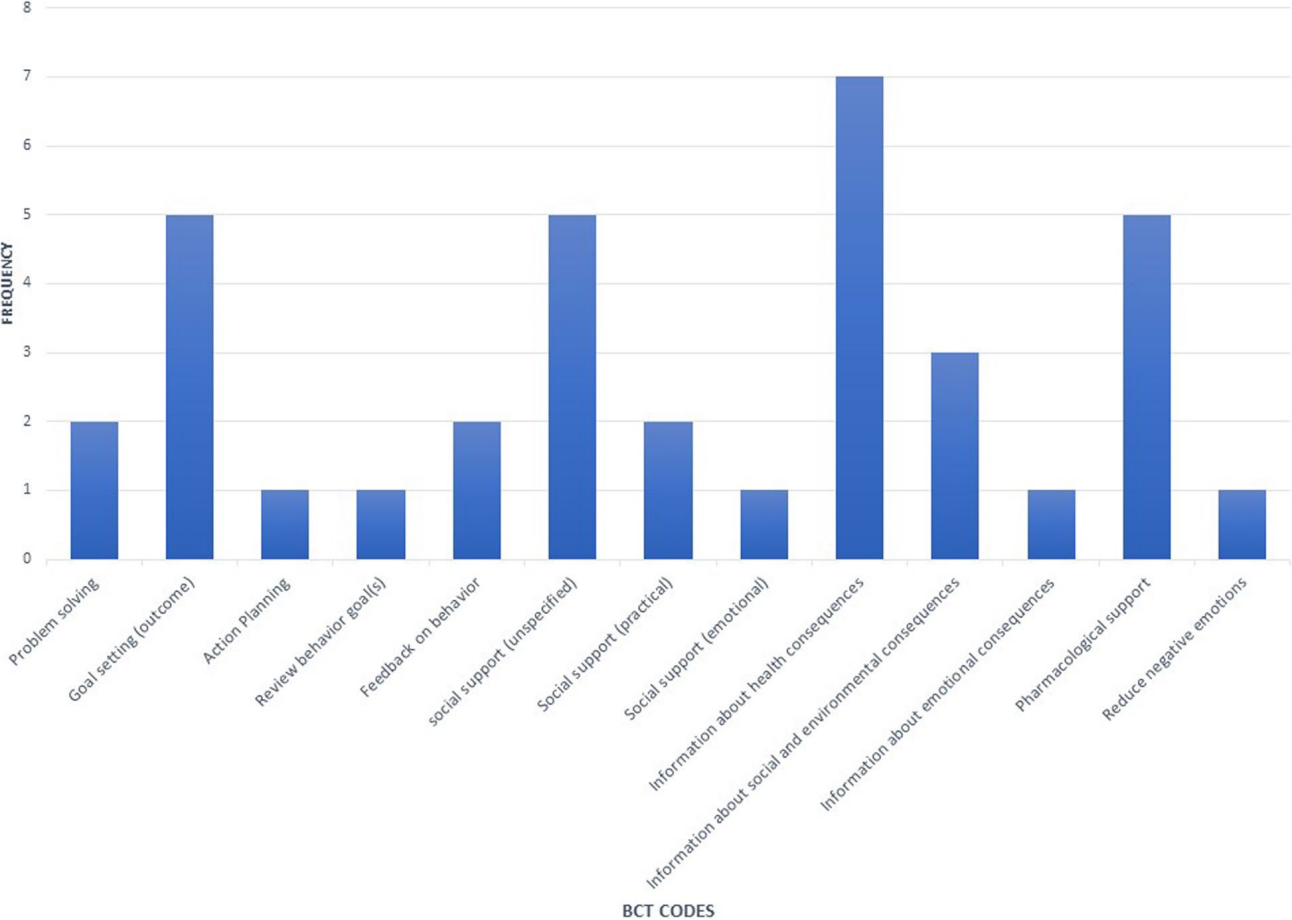
BCT analysis

Many of the interventions provided very little information on the details of the intervention content and the BCT codes that emerged from the interventions were narrow in focus (Fig. 2). Of the 93 active ingredients developed by Michie et al. [29], 13 different BCT codes were identified with an average of 3 BCTs per study. The number of BCTs used per study ranged from 1 to 7. Among these, information about health consequences was the most common ingredient (*n* = 7) followed by social support (unspecified) and goal setting- outcome, used by five of the interventions.

Most of the interventions concentrated mainly on provision of information along with pharmacological support. Though some of the interventions incorporated social support, this mainly meant the mothers were advised to help fathers quit smoking and establish a smoke free home, referral to a nearby community-based cessation services, family counselling and telephone support. Five studies offered an optional free NRT in the form of patches or gum [44, 46, 47, 51, 52]. The duration of free NRT supply offered was 1 week (*n* = 3) [46, 47, 52] and 6 weeks (*n* = 1) [51]. One of the studies did not specify the details of the NRT support provision [44].

### Outcome characteristics

All the studies used quit rates as a measure of success of the intervention and several studies used more than one outcome measure. The most common primary outcome reported was 7 days Point Prevalence (PP) of tobacco abstinence (*n* = 6) [44, 46–48, 50, 51] while others (*n* = 3) reported self-reported smoking status as primary outcome [45, 49, 52].

Smoking status was validated in all or at least a sample of the population at some time point in eight studies. Validation was done either by carbon monoxide (CO) reading (*n* = 3) [47, 48, 52], or saliva cotinine (*n* = 4) [44, 45, 50, 51] or both (*n* = 1) [46]. One of these studies used cotinine verification on children’s saliva sample [45] and another study [46], utilizing both the methods of verification used CO reading for validating the abstinence status of fathers triangulated by cotinine verification of the infant to confirm absence of SHS in infants. One single study [49] did not use any kind of biochemical verification but triangulated the abstinence data of fathers with self-reported exposure of SHS of mothers. Overall, only three studies reported their primary outcomes as validated abstinence [47, 48, 50].

In terms of outcome assessment period, most of the studies assessed the intervention outcome (at either or and) 3, 6, and 12 months after baseline [46–49, 51, 52]. McBride et al. [44] assessed their intervention at 28th week of pregnancy and at 2, 6 and 12 months postpartum, and Winickoff et al. [50] at 3 months after discharge from the hospital. One of the studies assessed outcome in the parents of the child when the child was 8 years old [45].

The outcome data were collected either through telephone interviews (*n* = 5) [46–48, 50, 52] using a detailed questionnaire, in person at health facility (*n* = 1) [45] or during home visits (*n* = 2) [49, 51]. One study [44] wasn’t explicit about the outcome data collection method.

Table 2 summarizes time points and the type of outcomes assessed in the included studies.

**Table 2:** Types and timepoints of outcomes assessed in the included studies

Study	Primary Outcome	Follow up time points (Primary outcome)	Secondary Outcomes
Chan et al., 2017 [46]	PP of father-reported tobacco abstinence in the past 7 days	6 months and 12 months	- biochemically validated abstinence, self-reported abstinence for at least 24 h (ie, quit attempt), reduction in daily cigarette consumption by at least 50% compared with baseline (ie, smoking reduction) - infant’s saliva cotinine concentrations at the 6 and 12 months follow-ups At 12 months follow-up: - mother reported tangible methods for helping the father and psychosocial support for the father in the past 6 months - mother reported fathers’ 6 months PP abstinence at the 12 months follow-up
Kallio et al., 2006 [45]	Parents reported smoking, reported child’s exposure to SHS	At the child’s age of 8 years	-SHS exposure of the child three consecutive days prior to the visit at their 8-yr-old visit
Luk et al., 2021 [47]	Biochemically validated tobacco abstinence at 6 months after intervention initiation	6 months	-self-reported 24 week continuous abstinence at 6 months after intervention initiation as well as 7-day PP abstinence -use of any NRT -use of a smoking cessation service at 3 and 6 months after intervention initiation
McBride et al., 2004 [44]	Self-reported smoking status in the past 7 days	28 weeks of pregnancy 2, 6, and 12 months postpartum	-Smoking-specific support assessed at baseline and each follow-up. Women and men independently completed a ten-item version of the Partner Interaction Questionnaire to assess positive and negative perceived and provided support for cessation -Men were asked the frequency with which they performed those behaviors (provided support)
Pollak et al., 2014 [51]	7-day PP abstinence	end of pregnancy (34 weeks gestation) 12 months post randomization	-30 day PP abstinence at the end of pregnancy (34 weeks gestation), and 12 months post randomization -Continuous abstinence at both follow-up timepoints
Stanton et al., 2004 [52]	Self-reported smoking status	End of pregnancy (approximately 6 months)	-
Winickoff et al., 2010 [50]	7-day PP of cotinine verified tobacco abstinence	3 months postpartum	-Percentage of parents who reported quit attempts that lasted 24 h in intervention and control time periods at the 3 months follow-up
Xia et al., 2020 [48]	Validated abstinence from smoking confirmed by a carbon monoxide level in expired air < 4 ppm	6 months follow-up	-self-reported 7-day PP abstinence -levels of readiness to quit at 6 months
Yu et al., 2017 [49]	-Self-reported smoking cessation among fathers -Self-reported exposure to household SHS among mothers of the newborns	6 and 12 months post-randomization	-fathers’ self-reported intent to quit, knowledge of SHS and tobacco smoking, and smoke-free home policy enforcement at the 6 and 12 months follow-up periods

### Cessation outcomes

Among the six studies reporting 7-day PP as primary or secondary outcome (Table 3), five showed positive effects in the abstinence rates (with most reporting statistically significant improvement) when compared to their control groups [44, 46–48, 50] and one resulted in marginal change [51] both at 6 months follow up (0.30 vs. 0.31 with adjusted OR 0.96 (0.60–1.55) (95% CI) and 12 months post randomization (0.38% vs 0.39% with adjusted OR -1.02 (0.65–1.60 (95% CI)) [51].

**Table 3:** Studies reporting 7-day PP as primary or secondary outcome

S.No	Study	7-day Point Prevalence Quit rates (Intervention Vs Control)		
		3 months	6 months (Intervention Vs Control)	12 months
1	Chan et al., 2017 [46]	-	13.4% vs 7.5% (OR, 2.10; 95% CI, 1.30–3.40; *P* < .01) (Self-reported)	13.7% vs 8.0% (OR, 1.92; 95% CI, 1.16–3.17; *P* < .01) (self-reported)
2	Luk et al., 2021 [47]	17.3 vs 12.4 (OR, 1.48; 95% CI, 1.05–2.09; *P* = .03) (self-reported)	26.4% vs 17.1% (OR, 1.74; 95% CI, 1.29–2.34; *P* < .001) (self-reported)	-
3	McBride et al., 2004 [44]	15% vs 5% X2 = 5.11, *p* = 0.02 (self-reported) At 28 weeks, corresponding to almost 3–4 months follow up	-	-
4	Pollak et al., 2014 [51]	-	0.30% vs 0.31% adjusted OR 0.96 (0.60–1.55), 95% CI at end of pregnancy, almost corresponding to 6 months (Cotinine verified)	0.38% vs 0.39%;OR (95% CI) = 1.02 (0.65–1.60) (Cotinine verified)
5	Winickoff et al., 2010 [50]	-Among fathers who smoked in the baseline, 7-day PP abstinence: 31% at baseline and 25% at follow up (intervention group) vs 38% at baseline and 23% at follow-up (control group) (effect size 9.4%; nonsignificant) (self-reported) -cotinine-confirmed 7-day abstinence 9% vs 3% (nonsignificant)		-
6	Xia et al., 2020 [48]		-Video group vs control: 25.7% Vs 11.4% *P* < 0.001 text group vs control: 17.4% versus 11.4%, *P* = 0.02 adjusted ORs were higher in the video intervention group (2.50, 95% CI: 1.65–3.80, *P* < 0.001) and text group (1.61, 95% CI: 1.04–2.50, *P* = 0.03) than in the control group -Video group also had significantly higher 7-day PP abstinence than the text group (24.6% vs 17.4%, *P* = 0.02), with an adjusted OR of 1.56 (95% CI: 1.07–2.29, *P* = 0.02) (Self-reported)	-

Two of the remaining three studies [49, 52] that did not report 7-day PP as primary or secondary outcome, also showed positive results. Yu et al. [49] reported abstinence rates of fathers at 6 and 12 months follow up showing a significant increase in the intervention group compared to the control. The abstinence rates (self-reported quitting) of father-focused intervention vs control at 6 months were (20.0% vs.7.3% control; adjusted odds ratio (OR):3.60, 95% CI: 1.41–9.25; *p* = 0.008). Smoking abstinence at 12 months was 22.7% in group I-B compared to 9.7% in the control group (adjusted OR: 2.93, 95% CI: 1.24–6.94; *p* = 0.014). Likewise, Stanton et al. [52] showed similar results at 6 months follow-up, with 16.5% of smoking partners in the intervention group and 9.3% in the control group having stopped smoking (*P* = 0.011, OR = 0.52, 95% CI, 0.31 – 0.86). Kallio et al. [45] on the other hand did not find effects in child’s exposure to tobacco smoke at eight years old using serum cotinine concentration. Meta-analysis of the outcome effectiveness wasn’t possible due to insufficient number of studies and heterogeneity in the type of interventions, thus not allowing to gain broader insights on concrete effectiveness of these interventions.

#### Couples vs fathers only interventions

The three studies evaluating interventions specifically among men [47, 48, 52] with no partner component demonstrated significantly higher smoking cessation rates than their control group. Studies that focused on men alongside intervention components addressing women had mixed effects on men’s smoking outcomes. While two studies by Chan et al. and Yu et al. showed that including female partners as a supportive aid in interventions is an effective way to increase male smoking cessation [46, 49], study by Pollak et al. reported little arm difference between more intensive couples-based counseling intervention as compared to provision of only culturally adapted written materials and NRT to expectant and postpartum fathers [51]. Two other studies by Kallio et al. and Winickoff et al. providing the same intervention to both partners without any distinction [45, 50] showed no significant differences in parental (and fathers’) smoking between the intervention and the control groups. Similarly, the study by McBride et al. focusing on women ‘s cessation with some partner component to men [44] showed no significant differences by condition in women’s reports of abstinence at any follow-up. However, this study noted significant increase in short-term cessation among partners in the partner-assisted intervention compared to the women-only intervention.

## Discussion

To our knowledge, this is the first review to summarize outcome effectiveness of behavior change focused smoking cessation interventions targeting expectant and new fathers.

Gender influences health and intersects with other social determinants of health both in shaping health behavior and contributing to positive health outcomes [54]. In many communities and settings, traditional masculine gender norms reinforce smoking in males. Although the implications of socio-cultural impact of gender roles on men’s smoking and cessation efforts are receiving increasing attention, this review highlights a lack of literature on smoking cessation interventions focusing specifically on expectant and new fathers. Our results confirm findings by Chizimo et al. [20] which identified 11 smoking cessation intervention studies specifically on men, including only one study among expectant fathers. Similar results were obtained in another study [37] reviewing perinatal partner smoking cessation interventions. They identified five studies reporting changes in male partners’ smoking status following a cessation intervention. Among these, only two studies had the main focus on partner cessation, one in the context of the family unit and the other solely aimed at the men. Nonetheless, these studies recognize smoking cessation among partners as an important component of maternal prenatal smoking cessation and are suggestive of supportive approaches to address partners’ needs to promote prenatal smoking cessation.

Gender roles have various dimensions and meaning in terms of shaping certain health behavior, and the concept of masculinity seems central for both defining smoking as well as cessation efforts [19]. Social constructionist view holds masculinity to be context-dependent, dynamic, fluid, and plural, constituted by social relations that produce identities entwined with power and class [55]. Cigarette smoking is considered a social reproduction of masculinity or declaration of masculine identity because smoking fulfills constructed manly ideals of risk-taking, neglect of self-health, and strength and toughness associated with dominant masculinity [55]. In the context of fatherhood, this masculine identity refers to the need to fulfil the roles of protector, caregiver and breadwinner for the family thereby motivating many expectant and new fathers to achieve and sustain smoking cessation [54]. Most of the smoking cessation interventions identified in this review did not address these broader factors shaping gender roles and norms for expectant and new fathers. This is congruent with the findings of Kodriati et al. [19] who argue for the need to design smoking cessation interventions informed by cultural context, and promoting aspects of masculinities that are protective against smoking throughout men’s course of life.

Very often, smoking during pregnancy is framed as the woman’s health problem and related smoking cessation interventions have long been designed to address only women’s smoking. Moreover, many of the interventions put the burden of implementing measures to protect children from secondhand smoke at home on women as well by advising them to “avoid” being around smoke and not necessarily exploring the need to involve smoking partners [56]. To ensure gender equitable smoking cessation services for all, a holistic approach needs to be taken also addressing diversity among couples. Considerations should be given to both same- and different-sex relationships in developing an inclusive health care system for smoking cessation, regardless of gender or sexual orientation.

### Variation in outcome measures and duration of outcome assessment

Though there were some differences in outcome measures used across studies, most of them used 6 months quit rates (7-day PP) as primary outcome measure. This is in line with the recommendation of the Russell standard, which recommends assessing prolonged prevalence/continuous abstinence at six months or 12 months after the quit date as a standard practice [57]. However, Russell standard also proposes using the 6 or 12 months quit rates combined with a biochemical test, using expired air carbon monoxide. The outcomes reported by the studies in this review at 6 months were a mix of self-reported and biochemically validated measures, with the majority of them being self-reported only. This has implications. First, it does not allow comparison between studies. Secondly, it demonstrates little consistency in reporting smoking cessation outcomes, and finally use of self-reported outcome impacts on the confidence in the conclusions of these studies.

#### Evidence of effectiveness

This review suggests that BCI’s have the potential to improve smoking cessation outcomes for expected and new fathers. However, evidence of effectiveness of interventions addressing fathers could not be established. Included studies were not designed to show effectiveness of interventions addressing fathers compared to gender-neutral interventions, rather focus on intervention treatment vs. no treatment. In the absence of adequate evidence from smoking cessation programs demonstrating conclusive effectiveness of men-specific smoking cessation interventions, insights could be drawn from other domains of health services regarding effectiveness of this approach. There are a few suggestions of benefit from other health programs pointing to the positive outcomes of “gender specific interventions” for men. One such example is from the meta-analysis of BCIs to increase men’s physical activity which demonstrated that BCI targeting men’s physical activity can be effective [58]. Similarly, mental health promotion programs specifically designed for men have also shown to be a promising approach to engage men and making positive changes in their lives [59]. Several other studies (particularly in the field of sexual and reproductive health and maternal and child health) have shown that health behavior interventions engaging expectant or new fathers either as a part of couple-focused interventions or as a male only targeted approach exhibit favorable outcomes [60, 61]. These can serve as a basis to bring gender specific interventions for expectant and new fathers in the context of tobacco control policies and practices.

Another central finding from this review is an indication that even though pregnancy and birth of a child present good opportunities to identify smoking parents and is an appropriate teachable moment to provide cessation assistance, addressing this without any gender considerations in the interventions might not yield the optimum results for successful cessation. Interventions that take into account gender differences, rather than being gender-neutral, demonstrate potential to attain positive cessation outcomes.

#### Underreporting of theories and BCTS

Several studies suggest theory-based BCIs to be effective in terms of changing health risk behavior. While many of these studies come from other health programs, some are also from smoking cessation [62–64]. Though many of the BCIs claim to be theory-informed or theory-based, the extent to which the theory has been used as a foundation for intervention development and delivery is questionable and should be read with caution. To address issues like this, Michie & Prestwich developed a reliable coding system that rated use of theory according to five categories: (i) is theory mentioned? Are the relevant theoretical constructs targeted? (iii) Are the relevant theoretical constructs measured? (iv) Are mediation effects tested? (v) Is theory refined? [65]. They argue that assessing the use of theory for intervention design and evaluation would allow research in this area to progress.

The studies identified in this review insufficiently presented theories underlying the intervention, thus not allowing to assess those core concepts. Where interventions were mentioned to be based on theories or models, they do not sufficiently explain how the said theory contributed to the design and content of the intervention. To be able to categorize any intervention as theory based, use of theory to develop the intervention content is vital. Further, absence of any gender or masculinity theories to inform intervention design also points to the extensive work that needs to be done to keep gender at the centre for smoking cessation interventions.

As with the case of description of BCTs used, the contents of the interventions are also underreported. This concern echoes finding from literature illustrating limitations in the standards of reporting interventions to change health-related behaviors, specifically, smoking cessation [66]. Inadequate details do not allow comparability and ability to synthesize findings and to understand gaps and inconsistencies between the outlined (or planned) intervention and the ones implemented. This limits the guidance for future intervention review and replication [29]. In areas like smoking cessation interventions among expectant and new fathers, where the evidence base on different BCIs are not strong, proper reporting of the details of the intervention by both the practitioners and research communities would enable to better understand the complexities and interrelationship between various principles of behavior change and potentially contribute to appropriate intervention designs.

#### Strengths and limitations

The strengths of this review include use of systematic methodology and broad scope of search terms to ensure wide range of BCIs coverage. The study employed a broad definition of BCI and encompassed a large spectrum of BCI strategies. This allowed inclusion of interventions which did not necessarily describe or classify themselves as BCIs. Some of the limitations of the study include exclusion of study designs other than RCTs. Since we aimed to examine the effects of BCI interventions on expectant and new fathers smoking behavior, we only considered randomized control design as RCT is considered to provide the most reliable evidence on the effectiveness of interventions. This may have led to exclusion of potential relevant studies using other study designs. Secondly, though we acknowledge gender as non binary, we focused our study on heterosexual couples. Still, we also used general search terms (e.g. “expectant/ new couples”, “expectant/new parents”) to capture expectant and new parent population which could include gender non-conforming individuals/couples. Finally, with limited information provided on intervention content in the identified studies, we assumed that interventions targeted to expectant and new fathers [6] were, at least to some extent, tailored to their needs.

## Conclusions

This review provides a useful synthesis of the current state of evidence related to effectiveness of BCIs for expectant and new fathers. Our findings clearly demonstrate lack of breadth of evidence needed to understand the effectiveness of these interventions on smoking cessation outcomes of expectant and new fathers. It also confirms the heterogeneity of studies conducted so far among expectant and new fathers, evidenced by varying definitions of BCI, interventions components, controls and outcome measures. Existing studies using BCIs insufficiently report details of intervention components, thus providing an incomplete picture of the range of intervention and BCT used so far Robust and transparent reporting of fatherspecific interventions and cessation outcomes could alleviate that.

### Supplementary Information

**Additional file 1:** **Supplementary material.** Search strings for different databases (Search 18 Nov 2021).

**Additional file 2: Supplementary Table.** Risk of Bias assessment (RoB) summary of the included studies.

## Data Availability

All data generated or analysed during this study are included in this published article [and its supplementary information files]. Any other datasets used and/or analysed during the current study are available from the corresponding author on reasonable request.

## Abbreviations

ANC: Antenatal Care
AWARD: Ask, Warn, Advise, Refer, and Do it again
BANSAR: Brief Advice, NRT sampling, and Active Referral
BCI: Behavior Change Intervention
BCT: Behavior Change Techniques
CO: Carbon monoxide
ETS: Environmental Tobacco Smoke
ENDS: Electronic Nicotine Delivery Systems
FCS: Family Counselling Session
FCTC: Framework Convention for Tobacco Control
MesH: Medical Subject Heading
MoD: Modes of Delivery
NRT: Nicotine Replacement Therapy
LMICs: Low- and Middle-Income Countries
PRISMA: Preferred Reporting Items for Systematic Review and Meta-Analysis
RCT: Randomised Control Trial
RoB 2: Revised Cochrane risk-of-bias tool
SDOH: Social Determinants of Health
SHS: Second-hand smoke
SGM: Sexual and Gender Minorities
WHO: World Health Organization
