# Supplementary material for: Effectiveness of behavior change interventions for smoking cessation among expectant and new fathers: findings from a systematic review
Source: BMC Public Health. 2023 Sep 18;23:1812. doi: 10.1186/s12889-023-16713-5 (PMC10506219; doi:10.1186/s12889-023-16713-5)
Supplement: Supplementary file 1 — Additional file 1: Supplementary material. Search strings for different databases (Search 18 Nov 2021). [file 12889_2023_16713_MOESM1_ESM.docx]

Supplementary material: Search strings for different databases (Search 18 Nov 2021)

1. ***PubMed***

N=96

(((((((("smokers"[MeSH Terms]) OR ("smokers")) OR ("smoking cessation"[MeSH Terms] OR "smoking cessation"[All Fields])) OR ("secondhand smoke"[All Fields] OR "passive smoking"[All Fields])) OR ("tobacco smoke pollution"[MeSH Terms] OR "tobacco smoke pollution"[All Fields])) OR ("Tobacco smoking" [MeSH Terms] OR "Tobacco smoking"[All Fields])) OR ("environmental tobacco smoke"[All Fields] OR "smoking abstinence"[All Fields]))

AND

(((((((((((((("counseling"[MeSH Terms] OR "counseling"[All Fields] OR "counselling"[All Fields]) OR ("cognitive behavioral therapy"[MeSH Terms])) OR ("brief therapy"[All Fields] OR "brief support"[All Fields] OR "brief advice"[All Fields])) OR ("psychotherapy, brief"[MeSH Terms] OR "psychotherapy, brief"[All Fields])) OR ("telephone support"[All Fields])) OR ("text messaging"[MeSH Terms] OR "text messag*"[All Fields])) OR ("Behavior Therapy"[MeSH Terms] OR "Behavior Therap*"[All Fields])) OR ("web-based intervention*"[All Fields] OR "internet based interventio*"[All Fields])) OR ("self-help materia*"[All Fields] OR "self-help resourc*"[All Fields])) OR ("pamphlets"[MeSH Terms] OR "pamphle*"[All Fields] OR "leaflet*"[All Fields])) OR ("audio tape*"[All Fields] OR "audiotape*"[All Fields] OR "videotape*"[All Fields] OR "video tape*"[All Fields])) OR ("audiovisual aids"[MeSH Terms] OR "audiovisual aids"[All Fields])) OR ("couples therapy"[MeSH Terms] OR "couples therapy"[All fields])) OR ("behavior change intervention"[All Fields] OR "behaviour change intervention"[All Fields])))

AND

((("fathers"[MeSH Terms] OR "father*"[All Fields] OR "spouses"[MeSH Terms] OR "spouse*"[All Fields] OR "coupl*"[All Fields] OR "partne*"[All Fields] OR "significant othe*"[All Fields]) AND ("pregnancy"[MeSH Terms] OR "pregnan*"[All Fields] OR "prenatal care"[MeSH Terms] OR "prenatal"[All Fields] OR "post-natal"[All Fields] OR "postpartum"[All Fields])) OR ("new fathe*"[All Fields] OR "expectant father*"[All Fields] OR "expecting father*"[All Fields] OR "new parent*"[All Fields] OR "expectant parent*"[All Fields] OR "expecting parent*"[All Fields]))

1. ***EMBASE***

N=133

((('smoking'/exp OR 'smoking') AND 'smoking':ti,ab,kw OR ('smokers' AND 'smokers':ti,ab,kw)) OR (('smoking cessation'/exp OR 'smoking cessation') AND 'smoking cessation':ti,ab,kw) OR (('passive smoking'/exp OR 'passive smoking') AND 'passive smoking':ti,ab,kw) OR ((('tobacco smoke pollution'/exp OR 'tobacco smoke pollution') AND 'tobacco smoke pollution':ti,ab,kw OR 'environmental tobacco smoke') AND 'environmental tobacco smoke':ti,ab,kw) OR (('tobacco smoking'/exp OR 'tobacco smoking') AND 'tobacco smoking':ti,ab,kw) OR (('secondhand smoke'/exp OR 'secondhand smoke') AND 'secondhandsmoke':ti,ab,kw) OR (('smoking abstinence'/exp OR 'smoking abstinence') AND 'smoking abstinence':ti,ab,kw))

AND

(('counseling'/exp OR 'counseling' OR 'counselling') OR ('cognitive behavioral therapy'/exp OR 'cognitive behavioral therapy') OR ('brief therapy' OR 'brief support' OR 'brief advice'/exp OR 'brief advice') OR ('psychotherapy, brief'/exp OR 'psychotherapy, brief') OR 'telephone support' OR ('text messaging'/exp OR 'text messaging' OR 'text messag*') OR ('behavior therapy'/exp OR 'behavior therapy' OR 'behavior therap*') OR ('web-based intervention'/exp OR 'web-based intervention' OR 'web-based interventio*' OR 'internet based interventio*') OR ('self help materia*' OR 'self help resource*') OR ('pamphle*' OR 'leafle*') OR ('audiotape*' OR 'audio tape*' OR 'videotape*' OR 'video tape*') OR ('audiovisual aid'/exp OR 'audiovisual aid' OR 'audiovisual aids') OR ('couple therapy'/exp OR 'couple therapy' OR 'couples therapy') OR ('behavior change intervention' OR 'behaviour change intervention'))

AND

(('expectant father'/exp OR 'expectant father' OR 'new fathe*' OR 'expecting fathe*' OR 'new parent*' OR 'expectant parent'/exp OR 'expectant parent' OR 'expecting parent*') OR (('father'/exp OR 'father' OR 'spouse'/exp OR 'spouse' OR 'couple'/exp OR 'couple' OR 'partner'/exp OR 'partner' OR 'significant other') AND ('pregnancy'/exp OR 'pregnancy' OR 'prenatal care'/exp OR 'prenatal care' OR 'prenatal' OR 'post natal' OR 'post partum')))

1. ***Wiley Online Library***

N=98

("smokers" OR "smoking cessation" OR "secondhand smoke" OR "passive smoking" OR "tobacco smoke pollution" OR "environmental tobacco smoke" OR "smoking abstinence" OR “Tobacco smoking”)" in Abstract

and

"(“counseling" OR "counselling" OR "cognitive behavioral therapy" OR "brief therapy" OR "brief support" OR "brief advice" OR "psychotherapy brief" OR "telephone support" OR "text messaging" OR "web based interventions" OR “internet based interventions” OR "self help materials" OR "self help resources" OR "pamphlets" OR "audio tapes" OR "audiotapes" OR "videotapes" OR "audiovisual aids" OR "leaflet*" OR "couples therapy" OR "behavior change intervention" OR "Behavior Therapy")" anywhere

and

"("fathers" OR "spouses" OR "new fathers" OR "expectant fathers" OR "expecting fathers" OR "pregnant couples")

1. ***JSTOR***

N=344

(((("smoking cessation" OR "cigarette smoking" OR "Tobacco smoking") AND ("counseling" OR "behavior NEAR 10 intervention")) AND ((("pregnan*") AND ("spouse*" OR "coupl*" OR "partne*")) OR "father*")))

Academic content: Journals

1. ***Web of Science Core Collection***

N=361

8

((#1) AND #2) AND #7

Edit

Add to Search

[361](https://www.webofscience.com/wos/woscc/summary/74c3c163-4432-4b71-a490-5db9b6acfddb-13ecf34f/relevance/1)

7

(#6) OR #4

Edit

Add to Search

[2,139,975](https://www.webofscience.com/wos/woscc/summary/c35f5448-0a64-46e6-a83f-0592639614c0-13ece82f/relevance/1)

6

(#4) AND #5

Edit

Add to Search

[38,558](https://www.webofscience.com/wos/woscc/summary/e1dc9b2e-7ff5-4ca8-b997-afd3a3d0bb83-13ecdf6c/relevance/1)

5

(ALL=("pregnancy" OR "pregnan*" OR "prenatal care" OR "prenatal" OR "post-natal" OR "postpartum" ))

Edit

Add to Search

[686,353](https://www.webofscience.com/wos/woscc/summary/1b63296e-20c3-49d8-830b-f762b382e03c-13ecd3d6/relevance/1)

4

(ALL=("fathers" OR "father*" OR "spouses" OR "spouse*" OR "coupl*" OR "partne*" OR "significant othe*" ))

Edit

Add to Search

[2,139,975](https://www.webofscience.com/wos/woscc/summary/56e3ff34-a5d5-44b2-bcc8-f493f62ea3a4-13ecce53/relevance/1)

3

ALL=("new fathe*" OR "expectant father*" OR "expecting father*" OR "new parent*" OR "expectant parent*" OR "expecting parent*")

Edit

Add to Search

[2,266](https://www.webofscience.com/wos/woscc/summary/457a3314-08b3-49d7-993d-c4087e4a77d6-13ecc6ff/relevance/1)

2

ALL=("counseling" OR "counselling" OR "cognitive behavioral therapy" OR "brief therapy" OR "brief support" OR "brief advice" OR "psychotherapy, brief" OR "telephone support" OR "text messag*" OR "behaviour therap*" OR "behaviour therap*" OR "web-based intervention*" OR " internet based intervention*" OR" self-help materia*" OR "self-help resourc*" OR "pamphle*" OR "leaflet*" OR "audio tapes" OR "audiotape*" OR "videotape*" OR "video tape*" OR "audiovisual aids" OR "couples therapy" OR "behavior change intervention")

Edit

Add to Search

[256,219](https://www.webofscience.com/wos/woscc/summary/dbd6652e-d8a2-4147-b14e-dcb20741d40f-13ecc097/relevance/1)

1

TS=("smokers" OR "smoking cessation" OR "secondhand smoke" OR "passive smoking" OR "Tobacco smoking" OR "tobacco smoke pollution" OR "environmental tobacco smoke" OR "smoking abstinence")

Edit

Add to Search

[119,738](https://www.webofscience.com/wos/woscc/summary/f3e34e06-50f9-484b-b544-a249dae26f39-13ecbaae/relevance/1)

1. ***APA PsycINFO***

N=38

S34

S10 AND S25 AND S33

**Expanders** - Apply equivalent subjects

**Search modes** - Boolean/Phrase

[**View Results**](javascript:__doPostBack('ctl00$ctl00$MainContentArea$MainContentArea$historyControl$HistoryRepeater$ctl00$linkResults','')) (38)

S33

S29 OR S32

**Expanders** - Apply equivalent subjects

**Search modes** - Boolean/Phrase

[**View Results**](javascript:__doPostBack('ctl00$ctl00$MainContentArea$MainContentArea$historyControl$HistoryRepeater$ctl01$linkResults','')) (12,941)

S32

S30 AND S31

**Expanders** - Apply equivalent subjects

**Search modes** - Boolean/Phrase

[**View Results**](javascript:__doPostBack('ctl00$ctl00$MainContentArea$MainContentArea$historyControl$HistoryRepeater$ctl02$linkResults','')) (11,817)

S31

( MM "Pregnancy" OR TX "pregnan*" ) OR ( MM "Prenatal Care" OR TX "prenatal" OR TX "antenatal" ) OR ( TX "post natal" OR TX "postpartum" )

**Expanders** - Apply equivalent subjects

**Search modes** - Boolean/Phrase

[**View Results**](javascript:__doPostBack('ctl00$ctl00$MainContentArea$MainContentArea$historyControl$HistoryRepeater$ctl03$linkResults','')) (86,678)

S30

( MM "Fathers" OR TX "fathe*" ) OR ( MM "spouses" OR TX "spous*" ) OR ( MM "couples" OR TX "coupl*" ) OR ( MM "partners" OR TX "partne*" ) OR ( MM "significant others" OR TX "significant othe*" )

**Expanders** - Apply equivalent subjects

**Search modes** - Boolean/Phrase

[**View Results**](javascript:__doPostBack('ctl00$ctl00$MainContentArea$MainContentArea$historyControl$HistoryRepeater$ctl04$linkResults','')) (259,018)

S29

S26 OR S27 OR S28

**Expanders** - Apply equivalent subjects

**Search modes** - Boolean/Phrase

[**View Results**](javascript:__doPostBack('ctl00$ctl00$MainContentArea$MainContentArea$historyControl$HistoryRepeater$ctl05$linkResults','')) (1,767)

S28

TX "new parent*" OR TX "expectant paren*" OR TX "expecting paren*"

**Expanders** - Apply equivalent subjects

**Search modes** - Boolean/Phrase

[**View Results**](javascript:__doPostBack('ctl00$ctl00$MainContentArea$MainContentArea$historyControl$HistoryRepeater$ctl06$linkResults','')) (1,226)

S27

MM "Expectant Fathers" OR TX "expectant fathe*" OR TX "expecting father*"

**Expanders** - Apply equivalent subjects

**Search modes** - Boolean/Phrase

[**View Results**](javascript:__doPostBack('ctl00$ctl00$MainContentArea$MainContentArea$historyControl$HistoryRepeater$ctl07$linkResults','')) (354)

S26

TX "new fathe*"

**Expanders** - Apply equivalent subjects

**Search modes** - Boolean/Phrase

[**View Results**](javascript:__doPostBack('ctl00$ctl00$MainContentArea$MainContentArea$historyControl$HistoryRepeater$ctl08$linkResults','')) (288)

S25

S11 OR S12 OR S13 OR S14 OR S15 OR S16 OR S17 OR S18 OR S19 OR S20 OR S21 OR S22 OR S23 OR S24

**Expanders** - Apply equivalent subjects

**Search modes** - Boolean/Phrase

[**View Results**](javascript:__doPostBack('ctl00$ctl00$MainContentArea$MainContentArea$historyControl$HistoryRepeater$ctl09$linkResults','')) (323,142)

S24

TX "behaviour change intervention" OR TX "behavior change intervention"

**Expanders** - Apply equivalent subjects

**Search modes** - Boolean/Phrase

[**View Results**](javascript:__doPostBack('ctl00$ctl00$MainContentArea$MainContentArea$historyControl$HistoryRepeater$ctl10$linkResults','')) (237)

S23

TX "couples therapy"

**Expanders** - Apply equivalent subjects

**Search modes** - Boolean/Phrase

[**View Results**](javascript:__doPostBack('ctl00$ctl00$MainContentArea$MainContentArea$historyControl$HistoryRepeater$ctl11$linkResults','')) (5,815)

S22

MM "Educational Audiovisual Aids" OR TX "Educational Audiovisual Aids" OR TX "audiovisual aids"

**Expanders** - Apply equivalent subjects

**Search modes** - Boolean/Phrase

[**View Results**](javascript:__doPostBack('ctl00$ctl00$MainContentArea$MainContentArea$historyControl$HistoryRepeater$ctl12$linkResults','')) (1,302)

S21

MM "Audiotapes" OR TX "Audiotapes" OR TX "Audio tapes"

**Expanders** - Apply equivalent subjects

**Search modes** - Boolean/Phrase

[**View Results**](javascript:__doPostBack('ctl00$ctl00$MainContentArea$MainContentArea$historyControl$HistoryRepeater$ctl13$linkResults','')) (1,561)

S20

TX self help materia* OR TX "self-help resources" OR TX "pamphlets" OR "leaflet"

**Expanders** - Apply equivalent subjects

**Search modes** - Boolean/Phrase

[**View Results**](javascript:__doPostBack('ctl00$ctl00$MainContentArea$MainContentArea$historyControl$HistoryRepeater$ctl14$linkResults','')) (2,219)

S19

MM "Text Messaging" OR TX ( text messaging and public health )

**Expanders** - Apply equivalent subjects

**Search modes** - Boolean/Phrase

[**View Results**](javascript:__doPostBack('ctl00$ctl00$MainContentArea$MainContentArea$historyControl$HistoryRepeater$ctl15$linkResults','')) (1,073)

S18

MM "Behavior Therapy" OR TX "Behavior Therapy"

**Expanders** - Apply equivalent subjects

**Search modes** - Boolean/Phrase

[**View Results**](javascript:__doPostBack('ctl00$ctl00$MainContentArea$MainContentArea$historyControl$HistoryRepeater$ctl16$linkResults','')) (70,804)

S17

TX "telephone support"

**Expanders** - Apply equivalent subjects

**Search modes** - Boolean/Phrase

[**View Results**](javascript:__doPostBack('ctl00$ctl00$MainContentArea$MainContentArea$historyControl$HistoryRepeater$ctl17$linkResults','')) (349)

S16

TX "brief psychotherapy"

**Expanders** - Apply equivalent subjects

**Search modes** - Boolean/Phrase

[**View Results**](javascript:__doPostBack('ctl00$ctl00$MainContentArea$MainContentArea$historyControl$HistoryRepeater$ctl18$linkResults','')) (6,295)

S15

TX "brief advice"

**Expanders** - Apply equivalent subjects

**Search modes** - Boolean/Phrase

[**View Results**](javascript:__doPostBack('ctl00$ctl00$MainContentArea$MainContentArea$historyControl$HistoryRepeater$ctl19$linkResults','')) (270)

S14

TX "brief support"

**Expanders** - Apply equivalent subjects

**Search modes** - Boolean/Phrase

[**View Results**](javascript:__doPostBack('ctl00$ctl00$MainContentArea$MainContentArea$historyControl$HistoryRepeater$ctl20$linkResults','')) (18)

S13

TX "brief therapy"

**Expanders** - Apply equivalent subjects

**Search modes** - Boolean/Phrase

[**View Results**](javascript:__doPostBack('ctl00$ctl00$MainContentArea$MainContentArea$historyControl$HistoryRepeater$ctl21$linkResults','')) (2,019)

S12

MM "cognitive behavioral therapy" OR TX ( "cognitive behavioral therapy" or "cbt" or "cognitive behavioural therapy" )

**Expanders** - Apply equivalent subjects

**Search modes** - Boolean/Phrase

[**View Results**](javascript:__doPostBack('ctl00$ctl00$MainContentArea$MainContentArea$historyControl$HistoryRepeater$ctl22$linkResults','')) (26,740)

S11

MM "Counseling" OR TX "Counseling" OR TX "counselling"

**Expanders** - Apply equivalent subjects

**Search modes** - Boolean/Phrase

[**View Results**](javascript:__doPostBack('ctl00$ctl00$MainContentArea$MainContentArea$historyControl$HistoryRepeater$ctl23$linkResults','')) (243,017)

S10

S1 OR S2 OR S3 OR S4 OR S5 OR S6 OR S7 OR S8 OR S9

**Expanders** - Apply equivalent subjects

**Search modes** - Boolean/Phrase

[**View Results**](javascript:__doPostBack('ctl00$ctl00$MainContentArea$MainContentArea$historyControl$HistoryRepeater$ctl24$linkResults','')) (44,301)

S9

TX "smoking abstinence"

**Expanders** - Apply equivalent subjects

**Search modes** - Boolean/Phrase

[**View Results**](javascript:__doPostBack('ctl00$ctl00$MainContentArea$MainContentArea$historyControl$HistoryRepeater$ctl25$linkResults','')) (1,315)

S8

TX "tobacco smoke pollution"

**Expanders** - Apply equivalent subjects

**Search modes** - Boolean/Phrase

[**View Results**](javascript:__doPostBack('ctl00$ctl00$MainContentArea$MainContentArea$historyControl$HistoryRepeater$ctl26$linkResults','')) (1,458)

S7

TX "tobacco smoke pollution"

**Expanders** - Apply equivalent subjects

**Search modes** - Boolean/Phrase

[**View Results**](javascript:__doPostBack('ctl00$ctl00$MainContentArea$MainContentArea$historyControl$HistoryRepeater$ctl27$linkResults','')) (1,458)

S6

TX "environmental tobacco smoke"

**Expanders** - Apply equivalent subjects

**Search modes** - Boolean/Phrase

[**View Results**](javascript:__doPostBack('ctl00$ctl00$MainContentArea$MainContentArea$historyControl$HistoryRepeater$ctl28$linkResults','')) (443)

S5

TX "secondhand smoke"

**Expanders** - Apply equivalent subjects

**Search modes** - Boolean/Phrase

[**View Results**](javascript:__doPostBack('ctl00$ctl00$MainContentArea$MainContentArea$historyControl$HistoryRepeater$ctl29$linkResults','')) (934)

S4

KW "smokers" OR TX "smokers"

**Expanders** - Apply equivalent subjects

**Search modes** - Boolean/Phrase

[**View Results**](javascript:__doPostBack('ctl00$ctl00$MainContentArea$MainContentArea$historyControl$HistoryRepeater$ctl30$linkResults','')) (22,724)

S3

MM "Tobacco Smoking" OR TX "Tobacco Smoking"

**Expanders** - Apply equivalent subjects

**Search modes** - Boolean/Phrase

[**View Results**](javascript:__doPostBack('ctl00$ctl00$MainContentArea$MainContentArea$historyControl$HistoryRepeater$ctl31$linkResults','')) (34,451)

S2

MM "Passive Smoking" OR TX "passive smoking"

**Expanders** - Apply equivalent subjects

**Search modes** - Boolean/Phrase

[**View Results**](javascript:__doPostBack('ctl00$ctl00$MainContentArea$MainContentArea$historyControl$HistoryRepeater$ctl32$linkResults','')) (1,088)

S1

MM "Smoking Cessation" OR TX "Smoking Cessation"

**Expanders** - Apply equivalent subjects

**Search modes** - Boolean/Phrase

[**View Results**](javascript:__doPostBack('ctl00$ctl00$MainContentArea$MainContentArea$historyControl$HistoryRepeater$ctl33$linkResults','')) (19,181)

1. ***Cochrane Central Register of Controlled Trials (CENTRAL) (accessed via university library)***

N= 59 (31 Cochrane reviews and 27 trials)

#1 "smokers" (Word variations have been searched) 16744

#2 MeSH descriptor: [Smokers] explode all trees 364

#3 "smoking cessation" (Word variations have been searched) 11121

#4 MeSH descriptor: [Smoking Cessation] explode all trees 4274

#5 "secondhand smoke" (Word variations have been searched) 326

#6 "passive smoking" 394

#7 MeSH descriptor: [Tobacco Smoke Pollution] explode all trees 314

#8 "tobacco smoke pollution" (Word variations have been searched) 330

#9 "environmental tobacco smoke" (Word variations have been searched) 218

#10 "smoking abstinence" (Word variations have been searched) 1342

#11 "tobacco smoking" (Word variations have been searched) 1638

#12 MeSH descriptor: [Tobacco Smoking] explode all trees 243

#13 #1 OR #2 OR #3 OR #4 OR #5 OR #6 OR #7 OR #8 OR #9 OR #10 OR #11 OR #12 20677

#14 MeSH descriptor: [Counseling] explode all trees 5783

#15 "counselling" (Word variations have been searched) 26867

#16 "counseling" 25744

#17 MeSH descriptor: [Cognitive Behavioral Therapy] explode all trees 9773

#18 "brief therapy" (Word variations have been searched) 171

#19 "brief support" (Word variations have been searched) 87

#20 "brief advice" (Word variations have been searched) 531

#21 MeSH descriptor: [Psychotherapy, Brief] explode all trees 1053

#22 "psychotherapy brief" (Word variations have been searched) 1103

#23 "telephone support" (Word variations have been searched) 875

#24 "text messaging" (Word variations have been searched) 4613

#25 MeSH descriptor: [Text Messaging] explode all trees 1042

#26 "web based intervention*" (Word variations have been searched) 1317

#27 "internet based intervention*" (Word variations have been searched) 883

#28 "self help materials" (Word variations have been searched) 346

#29 "self help resources" (Word variations have been searched) 37

#30 "pamphlets" 1700

#31 MeSH descriptor: [Pamphlets] explode all trees 929

#32 "audio tapes" (Word variations have been searched) 266

#33 "audiotapes" (Word variations have been searched) 799

#34 "videotapes" (Word variations have been searched) 3146

#35 "audiovisual aids" (Word variations have been searched) 499

#36 MeSH descriptor: [Audiovisual Aids] explode all trees 3899

#37 "leaflet*" (Word variations have been searched) 2596

#38 MeSH descriptor: [Couples Therapy] explode all trees 251

#39 "couples therapy" (Word variations have been searched) 369

#40 "behavior change intervention" (Word variations have been searched) 774

#41 MeSH descriptor: [Behavior Therapy] explode all trees 17863

#42 "Behavior Therapy" (Word variations have been searched) 27085

#43 #14 OR #15 OR #16 OR #17 OR #18 OR #19 OR #20 OR #21 OR #22 OR #23 OR #24 OR #25 OR #26 OR #27 OR #28 OR #29 OR #30 OR #31 OR #32 OR #33 OR #34 OR #35 OR #36 OR #37 OR #38 OR #39 OR #40 OR #41 OR #42 60419

#44 "new father" (Word variations have been searched) 10

#45 "expectant father" 15

#46 "expecting father" (Word variations have been searched) 40

#47 "new parent" (Word variations have been searched) 108

#48 "expectant parents" (Word variations have been searched) 55

#49 "expecting parents" (Word variations have been searched) 55

#50 #44 OR #45 OR #46 OR #47 OR #48 OR #49 72

#51 MeSH descriptor: [Fathers] explode all trees 202

#52 "fathers" (Word variations have been searched) 1887

#53 MeSH descriptor: [Spouses] explode all trees 377

#54 "spouses" (Word variations have been searched) 2344

#55 "couples" (Word variations have been searched) 11015

#56 "partners" (Word variations have been searched) 13951

#57 "significant other" (Word variations have been searched) 706

#58 #51 OR #52 OR #53 OR #54 OR #55 OR #56 OR #57 12984

#59 MeSH descriptor: [Pregnancy] explode all trees 23426

#60 "pregnancy" (Word variations have been searched) 64843

#61 MeSH descriptor: [Prenatal Care] explode all trees 1602

#62 "prenatal" (Word variations have been searched) 7784

#63 "antenatal" (Word variations have been searched) 5506

#64 "post natal" (Word variations have been searched) 522

#65 "post partum" (Word variations have been searched) 1960

#66 #59 OR #60 OR #61 OR #62 OR #63 OR #64 OR #65 67500

#67 #58 AND #66 3094

#68 #67 OR #50 3131

#69 #13 AND #43 AND #68 59

1. ***MEDLINE***

N=93

S39

S36 AND S37 AND S38

**Expanders** - Apply equivalent subjects

**Search modes** - Boolean/Phrase

[**View Results**](javascript:__doPostBack('ctl00$ctl00$MainContentArea$MainContentArea$historyControl$HistoryRepeater$ctl00$linkResults','')) (93)

S38

S9 OR S10 OR S11 OR S12 OR S13 OR S14 OR S15 OR S16 OR S17 OR S18 OR S19 OR S20 OR S21 OR S22 OR S23

**Expanders** - Apply equivalent subjects

**Search modes** - Boolean/Phrase

[**View Results**](javascript:__doPostBack('ctl00$ctl00$MainContentArea$MainContentArea$historyControl$HistoryRepeater$ctl01$linkResults','')) (277,288)

S37

S1 OR S2 OR S3 OR S4 OR S5 OR S6 OR S7 OR S8

**Expanders** - Apply equivalent subjects

**Search modes** - Boolean/Phrase

[**View Results**](javascript:__doPostBack('ctl00$ctl00$MainContentArea$MainContentArea$historyControl$HistoryRepeater$ctl02$linkResults','')) (130,852)

S36

S26 OR S35

**Expanders** - Apply equivalent subjects

**Search modes** - Boolean/Phrase

[**View Results**](javascript:__doPostBack('ctl00$ctl00$MainContentArea$MainContentArea$historyControl$HistoryRepeater$ctl03$linkResults','')) (41,966)

S35

S33 AND S34

**Expanders** - Apply equivalent subjects

**Search modes** - Boolean/Phrase

[**View Results**](javascript:__doPostBack('ctl00$ctl00$MainContentArea$MainContentArea$historyControl$HistoryRepeater$ctl04$linkResults','')) (40,410)

S34

S27 OR S28 OR S29 OR S30

**Expanders** - Apply equivalent subjects

**Search modes** - Boolean/Phrase

[**View Results**](javascript:__doPostBack('ctl00$ctl00$MainContentArea$MainContentArea$historyControl$HistoryRepeater$ctl05$linkResults','')) (592,920)

S33

S31 OR S32

**Expanders** - Apply equivalent subjects

**Search modes** - Boolean/Phrase

[**View Results**](javascript:__doPostBack('ctl00$ctl00$MainContentArea$MainContentArea$historyControl$HistoryRepeater$ctl06$linkResults','')) (1,135,859)

S32

(MM "Prenatal care") OR TX "prenatal*" OR TX "antenatal OR TX "post-natal" OR TX "post-partum"

**Expanders** - Apply equivalent subjects

**Search modes** - Boolean/Phrase

[**View Results**](javascript:__doPostBack('ctl00$ctl00$MainContentArea$MainContentArea$historyControl$HistoryRepeater$ctl07$linkResults','')) (244,633)

S31

(MM "Pregnancy") OR TX "pregnan*"

**Expanders** - Apply equivalent subjects

**Search modes** - Boolean/Phrase

[**View Results**](javascript:__doPostBack('ctl00$ctl00$MainContentArea$MainContentArea$historyControl$HistoryRepeater$ctl08$linkResults','')) (1,082,659)

S30

TX "partner" OR "significant other"

**Expanders** - Apply equivalent subjects

**Search modes** - Boolean/Phrase

[**View Results**](javascript:__doPostBack('ctl00$ctl00$MainContentArea$MainContentArea$historyControl$HistoryRepeater$ctl09$linkResults','')) (103,983)

S29

(MM "Spouses") OR TX "Spous*"

**Expanders** - Apply equivalent subjects

**Search modes** - Boolean/Phrase

[**View Results**](javascript:__doPostBack('ctl00$ctl00$MainContentArea$MainContentArea$historyControl$HistoryRepeater$ctl10$linkResults','')) (35,729)

S28

(MM "Couples") OR TX "couple*"

**Expanders** - Apply equivalent subjects

**Search modes** - Boolean/Phrase

[**View Results**](javascript:__doPostBack('ctl00$ctl00$MainContentArea$MainContentArea$historyControl$HistoryRepeater$ctl11$linkResults','')) (429,183)

S27

(MM "Fathers") OR "Father*"

**Expanders** - Apply equivalent subjects

**Search modes** - Boolean/Phrase

S26

S24 OR S25

**Expanders** - Apply equivalent subjects

**Search modes** - Boolean/Phrase

[**View Results**](javascript:__doPostBack('ctl00$ctl00$MainContentArea$MainContentArea$historyControl$HistoryRepeater$ctl13$linkResults','')) (2,169)

S25

TX "new parent*" OR TX "expectant parent*" OR TX "expecting parent*"

**Expanders** - Apply equivalent subjects

**Search modes** - Boolean/Phrase

[**View Results**](javascript:__doPostBack('ctl00$ctl00$MainContentArea$MainContentArea$historyControl$HistoryRepeater$ctl14$linkResults','')) (1,714)

S24

TX "new father*" OR TX "expectant father*" OR TX "expecting father*"

**Expanders** - Apply equivalent subjects

**Search modes** - Boolean/Phrase

[**View Results**](javascript:__doPostBack('ctl00$ctl00$MainContentArea$MainContentArea$historyControl$HistoryRepeater$ctl15$linkResults','')) (528)

S23

TX "behavior change intervention*"

**Expanders** - Apply equivalent subjects

**Search modes** - Boolean/Phrase

[**View Results**](javascript:__doPostBack('ctl00$ctl00$MainContentArea$MainContentArea$historyControl$HistoryRepeater$ctl16$linkResults','')) (1,243)

S22

(MM "Couples Therapy") OR TX "Couples Therapy"

**Expanders** - Apply equivalent subjects

**Search modes** - Boolean/Phrase

[**View Results**](javascript:__doPostBack('ctl00$ctl00$MainContentArea$MainContentArea$historyControl$HistoryRepeater$ctl17$linkResults','')) (1,088)

S21

(MM "Audiovisual Aids") OR "Audiovisual Aids"

**Expanders** - Apply equivalent subjects

**Search modes** - Boolean/Phrase

[**View Results**](javascript:__doPostBack('ctl00$ctl00$MainContentArea$MainContentArea$historyControl$HistoryRepeater$ctl18$linkResults','')) (7,202)

S20

TX "audio tape*" OR TX "audiotape*" OR TX "videotape*" OR "video tape*"

**Expanders** - Apply equivalent subjects

**Search modes** - Boolean/Phrase

[**View Results**](javascript:__doPostBack('ctl00$ctl00$MainContentArea$MainContentArea$historyControl$HistoryRepeater$ctl19$linkResults','')) (36,055)

S19

TX "leaflet*"

**Expanders** - Apply equivalent subjects

**Search modes** - Boolean/Phrase

[**View Results**](javascript:__doPostBack('ctl00$ctl00$MainContentArea$MainContentArea$historyControl$HistoryRepeater$ctl20$linkResults','')) (24,824)

S18

(MM "Pamphlets") OR TX "pamphlet*"

**Expanders** - Apply equivalent subjects

**Search modes** - Boolean/Phrase

[**View Results**](javascript:__doPostBack('ctl00$ctl00$MainContentArea$MainContentArea$historyControl$HistoryRepeater$ctl21$linkResults','')) (6,053)

S17

TX "self-help materia*" OR TX "self-help resourc*"

**Expanders** - Apply equivalent subjects

**Search modes** - Boolean/Phrase

[**View Results**](javascript:__doPostBack('ctl00$ctl00$MainContentArea$MainContentArea$historyControl$HistoryRepeater$ctl22$linkResults','')) (383)

S16

TX "web-based intervention*" OR TX “internet based intervention*”

**Expanders** - Apply equivalent subjects

**Search modes** - Boolean/Phrase

[**View Results**](javascript:__doPostBack('ctl00$ctl00$MainContentArea$MainContentArea$historyControl$HistoryRepeater$ctl23$linkResults','')) (3,165)

S15

(MM "Text Messaging") OR TX "Text Messag*"

**Expanders** - Apply equivalent subjects

**Search modes** - Boolean/Phrase

[**View Results**](javascript:__doPostBack('ctl00$ctl00$MainContentArea$MainContentArea$historyControl$HistoryRepeater$ctl24$linkResults','')) (7,253)

S14

TX "telephone support"

**Expanders** - Apply equivalent subjects

**Search modes** - Boolean/Phrase

[**View Results**](javascript:__doPostBack('ctl00$ctl00$MainContentArea$MainContentArea$historyControl$HistoryRepeater$ctl25$linkResults','')) (873)

S13

(MM "Psychotherapy, Brief") OR TX "Psychotherapy, Brief"

**Expanders** - Apply equivalent subjects

**Search modes** - Boolean/Phrase

[**View Results**](javascript:__doPostBack('ctl00$ctl00$MainContentArea$MainContentArea$historyControl$HistoryRepeater$ctl26$linkResults','')) (3,648)

S12

TX "brief therapy" OR TX "brief support" OR TX "brief advice"

**Expanders** - Apply equivalent subjects

**Search modes** - Boolean/Phrase

[**View Results**](javascript:__doPostBack('ctl00$ctl00$MainContentArea$MainContentArea$historyControl$HistoryRepeater$ctl27$linkResults','')) (1,049)

S11

(MM "Cognitive Behavior therapy")

**Expanders** - Apply equivalent subjects

**Search modes** - SmartText Searching

[**View Results**](javascript:__doPostBack('ctl00$ctl00$MainContentArea$MainContentArea$historyControl$HistoryRepeater$ctl28$linkResults','')) (180,566)

S10

(MM "Behavior therapy") OR TX "Behavior therap*"

**Expanders** - Apply equivalent subjects

**Search modes** - Boolean/Phrase

[**View Results**](javascript:__doPostBack('ctl00$ctl00$MainContentArea$MainContentArea$historyControl$HistoryRepeater$ctl29$linkResults','')) (38,320)

S9

(MM "Counseling") OR TX "counseling" OR TX "counselling"

**Expanders** - Apply equivalent subjects

**Search modes** - Boolean/Phrase

[**View Results**](javascript:__doPostBack('ctl00$ctl00$MainContentArea$MainContentArea$historyControl$HistoryRepeater$ctl30$linkResults','')) (156,286)

S8

(MM "Tobacco Smoke Pollution") OR TX "tobacco smoke pollution"

**Expanders** - Apply equivalent subjects

**Search modes** - Boolean/Phrase

[**View Results**](javascript:__doPostBack('ctl00$ctl00$MainContentArea$MainContentArea$historyControl$HistoryRepeater$ctl31$linkResults','')) (13,977)

S7

(MM "Tobacco Smoking") OR TX "tobacco smoking"

**Expanders** - Apply equivalent subjects

**Search modes** - Boolean/Phrase

[**View Results**](javascript:__doPostBack('ctl00$ctl00$MainContentArea$MainContentArea$historyControl$HistoryRepeater$ctl32$linkResults','')) (12,467)

S6

TX "smoking abstinence"

**Expanders** - Apply equivalent subjects

**Search modes** - Boolean/Phrase

[**View Results**](javascript:__doPostBack('ctl00$ctl00$MainContentArea$MainContentArea$historyControl$HistoryRepeater$ctl33$linkResults','')) (1,845)

S5

TX "environmental tobacco smoke"

**Expanders** - Apply equivalent subjects

**Search modes** - Boolean/Phrase

[**View Results**](javascript:__doPostBack('ctl00$ctl00$MainContentArea$MainContentArea$historyControl$HistoryRepeater$ctl34$linkResults','')) (3,978)

S4

TX "passive smoking"

**Expanders** - Apply equivalent subjects

**Search modes** - Boolean/Phrase

[**View Results**](javascript:__doPostBack('ctl00$ctl00$MainContentArea$MainContentArea$historyControl$HistoryRepeater$ctl35$linkResults','')) (4,359)

S3

TX "secondhand smoke"

**Expanders** - Apply equivalent subjects

**Search modes** - Boolean/Phrase

[**View Results**](javascript:__doPostBack('ctl00$ctl00$MainContentArea$MainContentArea$historyControl$HistoryRepeater$ctl36$linkResults','')) (3,160)

S2

(MM "Smoking cessation") OR TX "smoking cessation"

**Expanders** - Apply equivalent subjects

**Search modes** - Boolean/Phrase

[**View Results**](javascript:__doPostBack('ctl00$ctl00$MainContentArea$MainContentArea$historyControl$HistoryRepeater$ctl37$linkResults','')) (43,453)

S1

(MM "Smokers") OR TX "smokers"

**Expanders** - Apply equivalent subjects

**Search modes** - Boolean/Phrase

[**View Results**](javascript:__doPostBack('ctl00$ctl00$MainContentArea$MainContentArea$historyControl$HistoryRepeater$ctl38$linkResults','')) (89,494)
