# Supplementary material for: Effectiveness of behavior change interventions for smoking cessation among expectant and new fathers: findings from a systematic review
Source: BMC Public Health. 2023 Sep 18;23:1812. doi: 10.1186/s12889-023-16713-5 (PMC10506219; doi:10.1186/s12889-023-16713-5)
Supplement: Supplementary file 2 — Additional file 2: Supplementary Table. Risk of Bias assessment (RoB) summary of the included studies. [file 12889_2023_16713_MOESM2_ESM.docx]

Supplementary Table: Risk of Bias assessment (RoB) summary of the included studies

| **Studies** | **Randomization process** | **Deviations from the intended interventions** | **Missing outcome data** | **Measurement of the outcome** | **Selection of the reported result** | **Overall RoB assessment** |
| --- | --- | --- | --- | --- | --- | --- |
| Chan et al., 2017 | Low | Low | Low | Some Concerns | Low | Low |
| Kallio et al., 2006 | Some Concerns | Some Concerns | Low | Low | Some Concerns | Some concerns |
| Luk et al., 2021 | Low | Low | Low | Low | Low | Low |
| McBride et al., 2004 | Some Concerns | Some Concerns | Low | High | Low | High |
| Pollak et al., 2014 | Low | Low | Low | Low | Low | Low |
| Stanton et al., 2004 | Low | Low | Low | Low | Low | Low |
| Winickoff et al., 2010 | Low | Low | Low | Low | Low | Low |
| Xia et al., 2020 | Low | Low | Low | Low | Low | Low |
| Yu et al., 2017 | Low | Low | Low | High | Some Concerns | Some concerns |
